# An in-depth survey of the microbial landscape of the walls of a neonatal operating room

**DOI:** 10.1371/journal.pone.0230957

**Published:** 2020-04-03

**Authors:** Dieunel Derilus, Filipa Godoy-Vitorino, Hebe Rosado, Edgardo Agosto, Maria Gloria Dominguez-Bello, Humberto Cavallin

**Affiliations:** 1 Department of Environmental Sciences, University of Puerto Rico, San Juan, Puerto Rico, United States of America; 2 Department of Microbiology and Medical Zoology, University of Puerto-Rico-School of Medicine, San Juan, Puerto Rico, United States of America; 3 W. Harry Feinstone Department of Molecular Microbiology and Immunology, Bloomberg School of Public Health, Johns Hopkins University, Baltimore, Maryland, United States of America; 4 School of Architecture, University of Puerto Rico, San Juan, Puerto Rico, United States of America; 5 Department of Biochemistry and Microbiology, and of Anthropology, and the New Jersey Institute for Food Nutrition and Health, Rutgers University, New Brunswick, New Jersey, United States of America; University of Maine, UNITED STATES

## Abstract

Bacteria found in operating rooms (ORs) might be clinically relevant since they could pose a threat to patients. In addition, C-sections operations are performed in ORs that provide the first environment and bacterial exposure to the sterile newborns that are extracted directly from the uterus to the OR air. Considering that at least one third of neonates in the US are born via C-section delivery (and more than 50% of all deliveries in some countries), understanding the distribution of bacterial diversity in ORs is critical to better understanding the contribution of the OR microbiota to C-section- associated inflammatory diseases. Here, we mapped the bacteria contained in an OR after a procedure was performed; we sampled grids of 60x60 cm across walls and wall-adjacent floors and sequenced the V4 region of *16S rRNA* gene from 260 samples. The results indicate that bacterial communities changed significantly (ANOSIM, p-value < 0.001) with wall height, with an associated reduction of alpha diversity (t-test, p-value <0.05). OR walls contained high proportions of Proteobacteria, Firmicutes, and Actinobacteria, with Proteobacteria and Bacteroidetes being the highest in floors and lowest in the highest wall sites. Members of Firmicutes, Deinococcus-thermus, and Actinobacteria increased with wall height. Source-track analysis estimate that human skin is the major source contributing to bacterial composition in the OR walls, with an increase of bacteria related to human feces in the lowest walls and airborne bacteria in the highest wall sites. The results show that bacterial exposure in ORs varies spatially, and evidence exposure of C-section born neonates to human bacteria that remain on the floors and walls, possibly accumulated from patients, health, and cleaning staff.

## Introduction

Operating rooms (ORs) are spaces both designed and managed to minimize the negative impacts on the health of those that use them. As Parvizy et al. [1] pointed out, current guidelines place a significant focus on reducing environmental contamination of the ORs via cleaning and disinfection of hard and soft environmental surfaces, equipment, skin and hands of patients and health care workers. Other sources of contamination include limiting door openings and the number of individuals in the room during an intervention. Air related controls, as maintaining positive air pressure, air recirculation, use of HEPA air filters and controls for both temperature and humidity [2]. Several studies have already reported bacterial presence in C-section ORs using culture-dependent methods, pulse-field gel electrophoresis, fluorescent particle counting, and adenosine triphosphate (ATP) testing [3–6]. As humans lack natural ventilation during their stay in operating rooms, and, regardless of the efficacy of cleaning and the use of filtered air, they are expected to shed skin bacteria and thus enriched the environment with human bacteria [3,7–9].

Humans shed up to 37 million bacterial genomes into the environment per hour [10]. It has been shown that C-section babies have a higher abundance of skin-like bacteria (*Staphylococcus*, *Corynebacterium*, and *Propionibacterium*) at birth [11], which can also be acquired from the OR [3]. Previous studies using culture-dependent methods also showed that over 85% of air samples from OR's had skin-like bacteria, which were mostly coagulase-negative *Staphylococci* and *Corynebacterium* [3]. Other studies have shown that ORs have airborne skin bacteria that accumulate on surfaces [5,12,13].

Children born via C-section compared to those born vaginally are more likely to develop immune-related disorders and chronic diseases such as asthma/allergies [14], IBD [15], or obesity [16]. The changes associated to the differential microbiome seeding of newborns, in the case of those born via C-section likely, have an OR-factor, namely from the surrounding walls of the room [5]. Understanding the detailed composition of the OR walls may shed some light on the particular microbes that colonize newborns and may be relevant to newborn health, in societies of increased indoor lifestyles and with higher immune and metabolic disorders.

Because of the relevance that these built-environment conditions at the OR pose for newly born infants, we generated a map of the bacteria located on the wall surfaces of an operating room. The results will help better understand the association between increased risk of immune diseases observed in C-section born infants [17,18], increased risk for immune diseases.

## Materials and methods

### Sampling procedures

Samples were collected from an Operating Room immediately following C-section procedures at a public hospital in San Juan, Puerto Rico. The identities of the specific facilities were kept anonymous at the request of the established Institutional Review Board protocol agreement of the University of Puerto Rico, # 1112–172, and we had written authorization to collect samples from the Hospital.

We obtained dust from 260 samples, corresponding to four walls and-adjacent floors in this OR, by systematically subdividing the walls into 60x60 cm squares at four vertical distance levels measured at the floor level (0 cm. (“0”); and three (3) measurements on the wall, named 30 cm (“30”), 90 cm (“90”) and 150 cm (“150”) that correspond to the distance from the floor measured to the center of each of those squares and distributed horizontally at every 30 cm for each of the four walls (See S1 Data of S1A Fig). Environmental samples were obtained with sterile dry cotton swabs with wood shafts (Fisherbrand Catalog No. 23-400-114), by swabbing diagonally across the full 30 cm by 30 cm squared area determined for each sampling point.

Approximately 80% of the total sampled surface was covered by laminated plastic (walls 1, 2 and 4), and mostly glass in wall 3. In the wall areas that had posters, charts, and/or removable equipment, samples were taken from the wall behind those objects. After sampling, the swabs were refrigerated using liquid nitrogen and stored at −80°C until genomic DNA extraction.

### Genomic DNA extraction and sequencing

Total DNA was extracted using the MoBio (CA, USA) PowerSoil®-htp 96 Well Soil DNA Isolation plates according to the manufacturer's procedure. The V4 region of *16S rRNA* gene was amplified using the barcoded universal prokaryotic primer set 515F/806R [19], targeting both Archaea and Bacteria (Illumina MiSeq platform, using kit V3 300 which was run as paired end 150).

### Data analysis

#### Raw read pre-processing, filtering, and demultiplexing

The *16S rRNA* paired-end reads were checked for quality control using FastQC software [19], and the pair of forward and reverse reads were merged using FastQC-join method in QIIME following de default parameters. The resulting merged reads were further filtered and demultiplexed by the following criteria: minimum sequence length = 200 bp, and maximum numbers of ambiguous bases = 6. After filtering and demultiplexing, from a total of 131 samples that were evenly distributed through four sample categories (wall height at 0, 30, 90 and 150 cm), a total of 1,450,310 good-quality reads were retained for downstream analyses. We only used samples with more than 1,400 reads, considering this was the lowest number of reads (S1 Data of S2 Table). The joined (200bp) reads were uploaded to QIITA ID 11897.

#### OTU picking and taxonomic assignment

The resulting filtered and demultiplexed reads were clustered in operational taxonomic units (OTUs) using the closed reference strategy based on a minimum similarity threshold of 97% to SILVA *16S rRNA* databases, which were pre-clustered at 97% identity [20]. The taxonomy assignment of the OTUs was also carried out against the SILVA *16S rRNA* database (version 132) using the default parameters of the closed-reference OTU picking workflow in QIITA that used QIIMEq2 1.9.1 [21]. The OTU picking process generated an OTU table containing the count of the number of sequences for each OTU, and taxonomy assignment for each individual sample. OTUs that were observed fewer than three times, as well as those matching to chloroplast, mitochondria, and unassigned taxa, were filtered out from the OTU table. The total number of *16S rRNA* filtered reads and OTUs per samples, and according to the metadata category (distance to the floor) were reported in S1 Data of S2 Table.

#### Alpha diversity

Alpha diversity of the bacterial communities through the indoor OR architecture (across the four walls) was performed by computing multiple rarefactions of the OTU table, which was subsampled without replacement at each sampling depth, with a maximum of 1,400 reads per sample. Rarefaction curves describing the number of observed OTUs as a function of sequencing effort were further plotted in QIIME 1.9.1 [22]. Species richness across all the samples was also estimated using Fisher's alpha diversity index, which assumes that the abundance of species follows the log series distribution [23]. Scattered box plots showing the species richness for each individual sample (grouped in category) was computed using the *Microbiomeanalyst* platform (https://www.microbiomeanalyst.ca) [24] To test the statistical significance of the species richness between any pair of sample categories, a non-parametric t-test with and the Bonferroni correction methods [25] was performed by using the default number of Monte Carlo permutations (999).

#### Beta diversity

Beta diversity was measured on the subsampled OTU table by using Bray-Curtis as well as weighted and unweighted UniFrac metrics in QIIME. Global beta diversity depicting the level of dissimilarity between samples and the group of samples was visualized using Principal coordinate analysis (PcoA) and NMDS in R using package (https://www.R-project.org). Additionally, the statistical significance of the dissimilarity between different sample categories (sampling height), was performed with the non-parametric statistical test ANOSIM (analysis of similarity) in QIIME. Specific tests for differences in the spread (dispersion, variability) among groups were done using PERMDISP with 999 permutations, in QIIME.

#### Identifying differentially abundant taxa

Analysis of variance (ANOVA) was used to test the OTUs that were significantly abundant in the different sample group categories (OR wall sampling height) in QIIME. OTUs exhibiting significant differential abundance (P-value<0.05) between group categories after FDR (False Discovery Rate) correction, were positively filtered from the original OTU table. Additional analyses were performed using Microbiomeanalyst platform (https://www.microbiomeanalyst.ca) using the original OTU table downloaded from QIITA. Significant differences in microbiota at the Phyla and genus/species levels were found using linear discriminant analysis for effect size (LEfSe) algorithm, identifying features that are statistically different among biological classes (Kruskal-Wallis sum-rank test, p<0.05) and an additional test to assess whether these differences are consistent with respect to expected biological behavior (Wilcoxon ran-sum test, p<0.05), expressed as linear discriminant analysis (LDA) values, shown as boxplots.

#### Microbial source tracking

Source tracking was performed via closed reference OTU picking on our OR samples, as well as those samples which were downloaded from SRA. After OTUs were picked from all samples using the same exact methodology, OTU tables were merged into a single OTU table used within sourcetracker [26]. SRA samples included human skin samples, under accession number PRJNA314604 [27] human cervicovaginal samples with accession number PRJNA429969 [28]; human gut samples with accession number PRJEB26004 [29] and air samples were downloaded from Meadow et al. [30]

#### Availability of supporting data

The raw sequences supporting the results of this article are available in QIITA project ID 11897 and the European Nucleotide Archive EBI Study: PRJEB34320. http://www.ebi.ac.uk/ena/data/view/ERP117205. Accession: PRJEB34320, SRA:ERP117205 BioProject:PRJEB34320; UCSDMI:qiita_sid_11897. Supplementary information is included with the article and available on the PLoS ONE website. The O.T.U table was provided as S1 Table.

## Results and discussion

**Fig 1:**
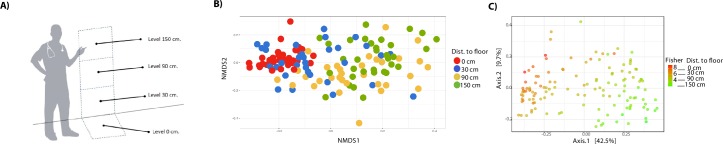
Detailed sampling diagram and beta diversity plots. Panel A is a diagrammatic description of the sampling panels at the OR done vertically. Panel B shows a non-metric multidimensional scaling (nMDS) plot, where points represent bacterial communities in samples of the OR, where walls are colored according to distance from the floor. Analysis of similarity (ANOSIM) on Bray-Curtis dissimilarity distance, showed a significant clustering (R = 0.04; *P* = 0.001). The statistical test for multivariate dispersions shows significant differences between communities at 0 cm, compared to all other wall heights (*P* = 0.001). Panel C shows a PCoA plot where samples are colored according to Fisher’s alpha diversity, with alpha diversity being higher among floor (0 cm) samples and lower among those at higher height (150 cm).

The V4 hypervariable region of bacterial *16S rRNA* genes (~254 bp) was amplified from metagenomic DNA obtained from surface swabs sampled at four vertical distance points (0, 30, 90 and 150 cm) for the four walls and-adjacent floors in an operating room (OR) (Fig 1, S1 Data of S1 Fig).

The resulting amplicon sets were sequenced using Illumina MiSeq paired-end-sequencing. After filtering and demultiplexing, from a total of 131 samples that were evenly distributed through four sample categories (wall height), a total of 1,450,310 good-quality reads were retained for downstream analyses (Table 1). A significant decrease in the average reads and OTUs close to the floor to the uppermost level of the OR walls level was observed (Table 1). This suggests that dust may have settled at lower surfaces as a result of gravity, and cooled air particles moved down through air convection.

 

**Table 1:** Summary statistics of the 16S rRNA genes bacterial reads across samples.

Sample Category (wall height distance from the floor)	Number of samples	Total reads	Number of read/samples (average-±stdev)	Total OTUs	Number of OTUs/sample (average-±stdev)
0 cm	33	543,859	16,481 (-±7,067)	4,516	557(-±217)
30 cm	33	432,102	10,605 (-±7,580)	3,460	316(-±160)
90 cm	32	257,210	8,038 (-±4,665)	2,773	263(-±110)
150 cm	33	17,139	6,580- (±3,183)	2,243	200(-±71)

Summary statistics of the 16S rRNA genes bacterial reads across the 131 samples organized by sample category (wall height). The number of samples, total reads, total OTUs, by sampling category (sampling height) are provided. The average number of reads and OTUs per sample/category are also provided. Both total number of 16S rRNA bacterial reads and the total number of OTUs decrease importantly with sampling height.

Beta diversity analyses show that bacterial communities cluster significantly by sampling height (Fig 1B). Analysis of similarity (ANOSIM) on Bray-Curtis dissimilarity distances, showed a significant clustering, especially separating samples at the floor level 0cm from the rest (R = 0.40; *P* = 0.001). Interestingly, the level of dissimilarity between groups of samples increases with their physical distance from the floor. This suggests that the microbiome of the floor samples resembles more to each other than the microbiome of the walls. Additionally, Principal Component Analyses with overlay Fisher alpha diversity levels show a higher diversity among floor-level samples compared to those at other wall heights separated along the first axis (Fig 1C). The test employed for multivariate dispersions, shows significant differences between communities at 0 cm, compared to all other wall heights (*P* = 0.001). The only non-significant comparisons were those between 30-90cm and 90-150cm (S1 Data of S1 Table).

**Fig 2:**
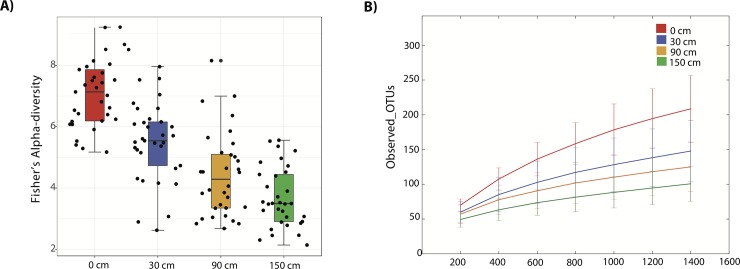
Comparison of alpha diversity at different sampling height of the OR for all walls combined. Panel A shows Fisher’s alpha diversity for different samples (black circles) through the four sampling height categories (Colored boxes). Diversity values were significant among wall height (*P* = 2.0048e-21; F-value: 50.06). Panel B shows the Rarefaction curves of observed OTUs by sampling category with a maximum sequencing depth of 1400 reads. *P*-values based on Monte Carlo permutations are depicted in the table inside the rarefaction curves.

Regarding alpha diversity and richness, both Fisher's alpha diversity metrics (Fig 2A) and rarefaction curves conducted at a maximum sequencing depth of 1400 reads (Fig 2B), reveal that both the bacterial diversity and richness decrease significantly with sampling height (*P* = 2.0048e-21; F-value: 50.06). This confirms the alpha diversity overlay in the PCoA plots, showing higher diversity in the floor samples. Conversely, to the samples close to the floor (0 cm), all the samples from the OR walls (30 cm, 90 cm, and 150 cm) were close to plateauing. This indicates that the maximum sequencing depth elected was reasonable, but an increase of this maximum value could contribute to increasing the number of OTUs, specifically in floor samples. The bacterial richness of the four walls was computed separately at the species level, finding that diversity shows an inverse proportion regarding sampling height (S1 Data of S2 Fig) that is consistent with the values for the OR with the always combined as shown in Fig 2. Overall, floor-level bacterial communities were significantly more diverse than those at the higher wall levels (S1 Data of S3 Fig).

**Fig 3:**
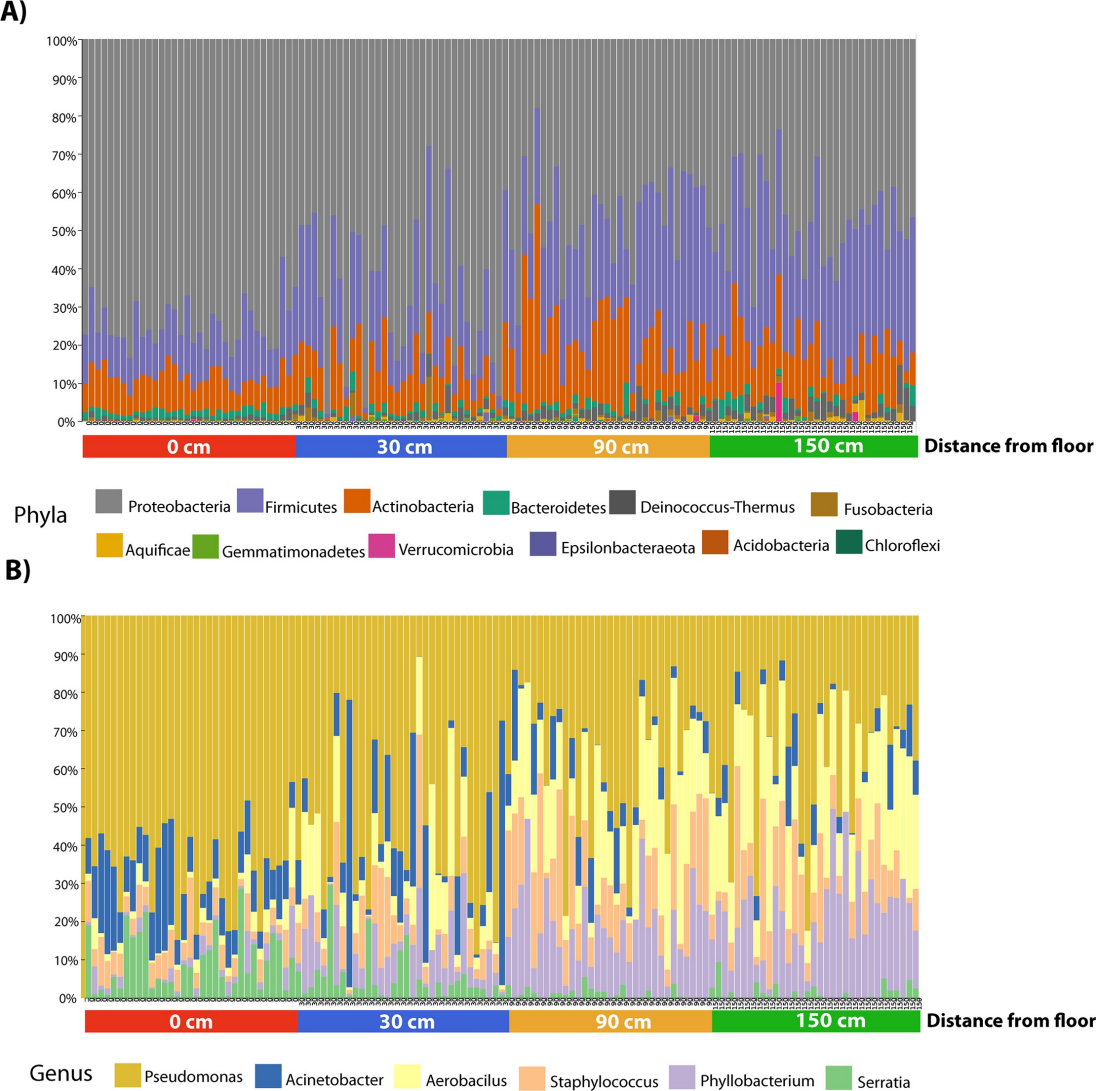
Taxonomy bar-plots. Panel A shows the relative abundance of the most abundant taxa at phyla, and Panel B show genus levels of the OR microbiota for different samples grouped in the sampling category. The genus-level plot was generated after filtering out all OTUs with minimum total observation fraction lower than 1%.

**Fig 4:**
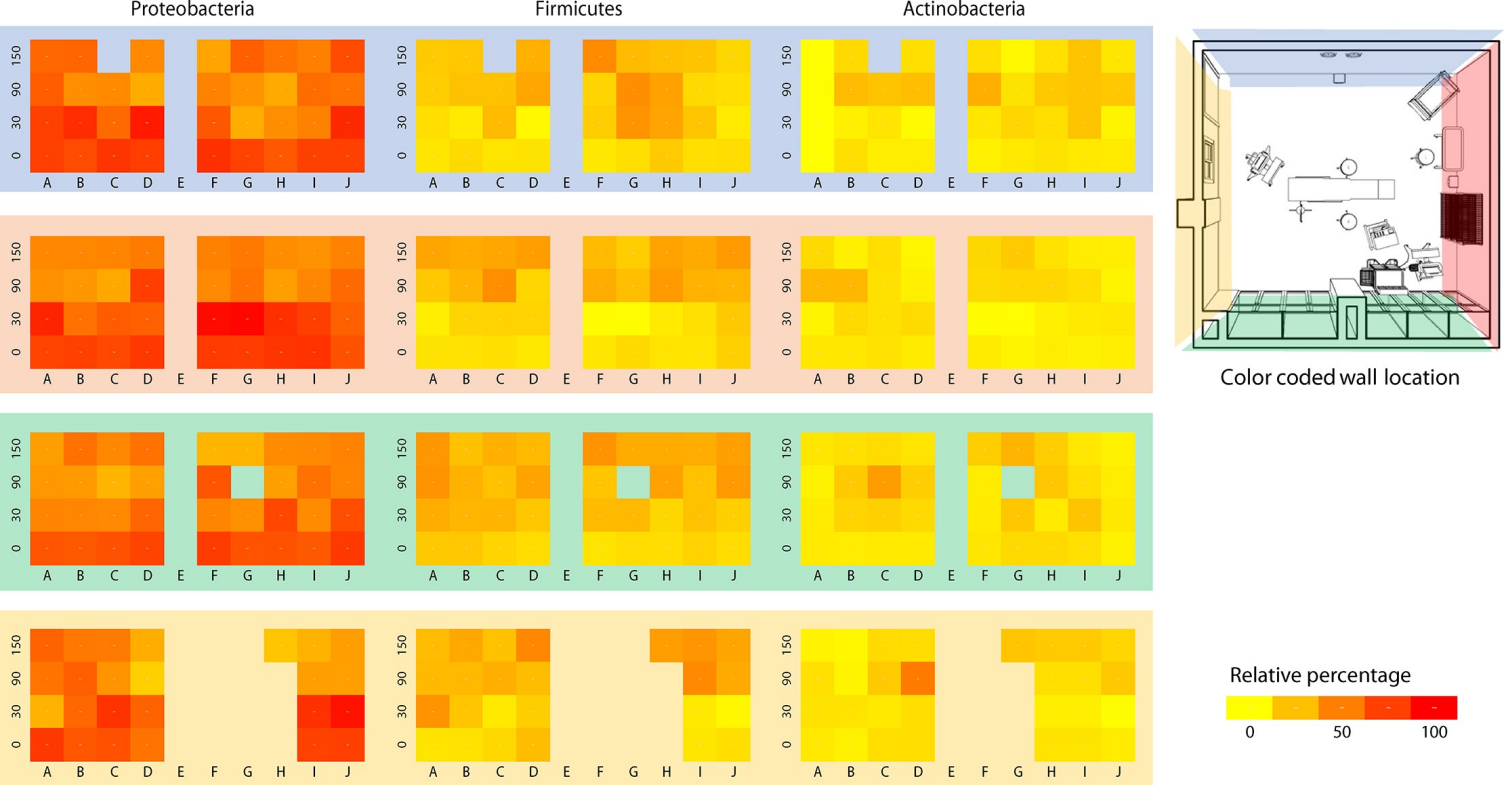
Representation of the relative abundance of bacteria from the most significant phyla on the four walls of the operating room. Each wall was subdivided into squares of 60 centimeters selected or swabbing, generating four rows that correspond to the levels 0 cm (floor), 30 cms, 90 cms, and 150 cms (measured from floor level to the center of each square). Each wall has been colored for reference, as seen in the top view of the operating room, as shown. We can see here the proportional variation connected to height variation for these walls, with a higher proportion of Proteobacteria the closer we get towards the level 0 cm (distant from the floor), and a proportional increment of both Firmicutes and Actinobacteria when we get farther away from the floor level.

**Fig 5:**
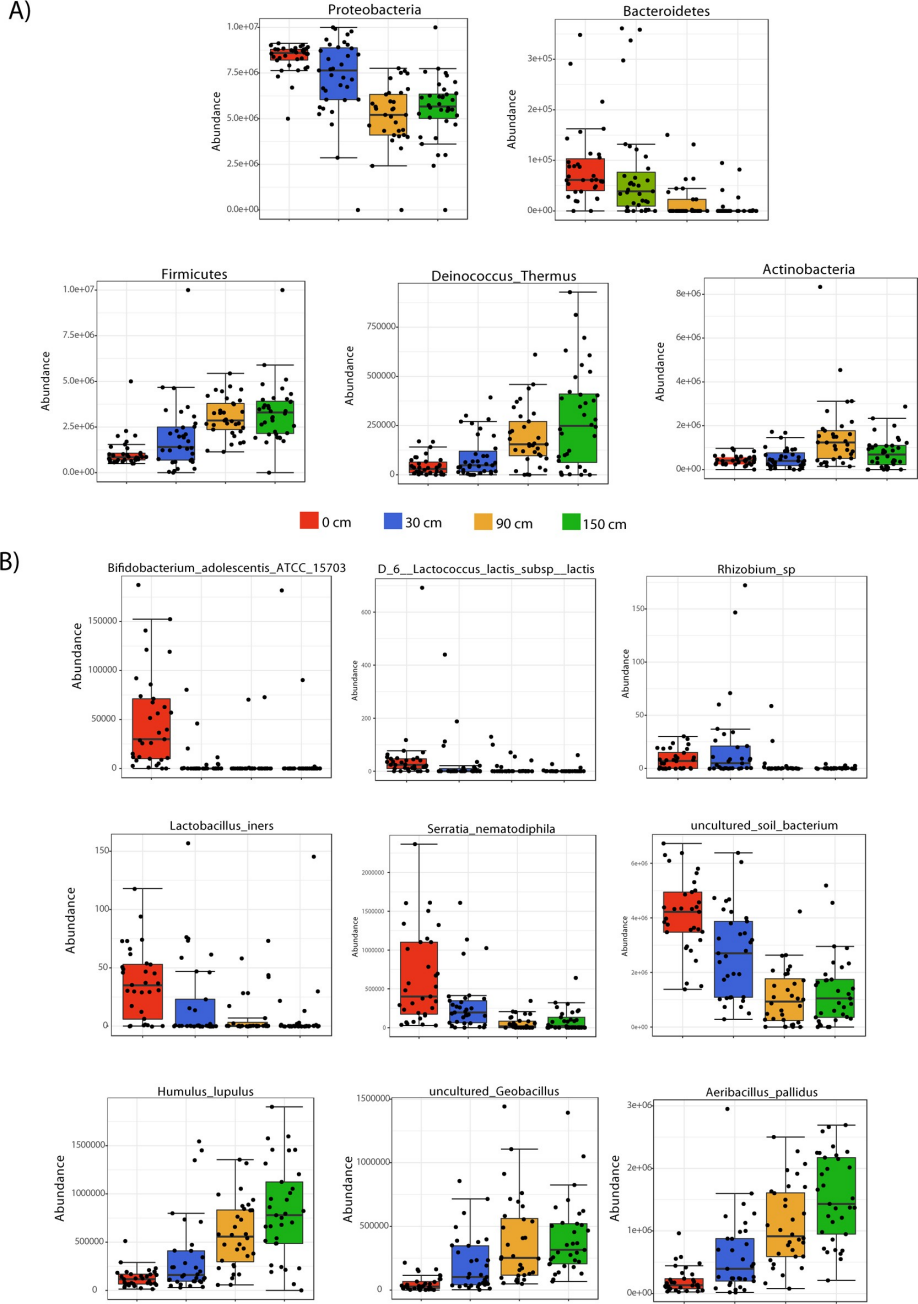
Differences in Phyla and genus levels between the wall heights of an OR. Differentially represented Phyla (panel A) and genus (panel B) showcasing differences between the wall heights of an OR. Analyses were done with LEfSe (Linear discriminant analysis Effect Size) algorithm by employing a non-parametric Kruskal-Wallis rank sum test and only taxa with FDR p-values <0.01 were plotted.

Regarding composition, the taxonomic assignment of the OTUs reveals that bacterial communities colonizing OR walls were predominantly from members of Proteobacteria, Firmicutes (most dominant at the floor level), and Actinobacteria, represent the three most dominant phyla (Fig 3A, Fig 4). We also found that also a relative abundance of five Phyla changed significantly with wall height (P-value<0.05); Proteobacteria and Bacteroidetes are more abundant in the floor and proportionally decrease with altitude, while Firmicutes and Deinococcus-Thermus increased proportionally with wall height (Figs 4 and 5).

At the genus level, members of *Pseudomonas*, *Acinetobacter*, *Aeribacillus*, *Staphylococcus*, *Phylobacterium* and *Serratia* were the most dominant genus in the OR walls (Fig 3B). The relative abundance of *Serratia*, *L*. *iners*, *B*. *adolescentis*, *Lactococcus lactis* were observed to be significantly more abundant at the floor (0 cm) levels (FDR p-values <0.01, Fig 5B), while members of *Humulus lupulus*, *Geobacilus* and *Aeribacillus pallidus* were more abundant with wall height (Fig 5B). The dominant populations at the floor level, indicate a soil, gut and vaginal origin of this bacteria, that is, taxa that are likely transported with shoes–like *Serratia*, a common soil and plant-associated bacteria [31] hat represents a nosocomial pathogen [32] or shed during C-sections (as suggested by the dominant *Lactobacillus* and *Bifidobacterium* [33]*—*accumulate at the soil level and decrease with wall height, while skin bacteria and other aerobic bacteria are more dominant with wall height, which might point out also to a relationship of access to both touching and cleaning the walls.

**Fig 6:**
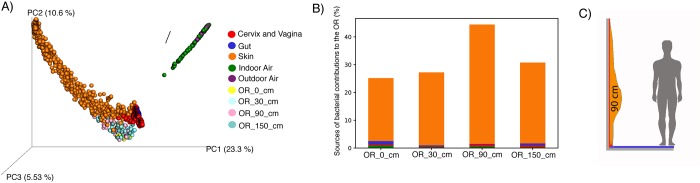
Source-tracking analyses. Panel A shows the PCoA with OR samples compared to other samples of the public repositories, including the cervix and vagina, gut, skin, and indoor and outdoor air. The bar plots of panel B show the relative contributions from the different sources for each sampled distance. Panel C summarizes the sampling conducted and shows higher abundances of skin microbes at 90cm.

Source-tracking analyses indeed revealed ground-level walls to be closer to gut microbes (Fig 6A, 6B and 6C), while at 90 cm the majority were skin-originating microbes (Fig 6C) and at 150 cm-level samples grouped closer to indoor and outdoor air microbes. An outlier of OR samples clustered even closer to air/environmental microbes, which corresponded to samples close to a window (Fig 6A, 6B and 6C).

In recognition of the impact of the built environment on the colonization of the newborn microbiome, we characterized the microbiota across the height of OR walls. Our results reveal that the studied post-C-section OR conceals an impressive array of bacteria that are unevenly distributed through the vertical dimension of the sampled walls. Results point out to potentially relevant associations between the location of the sample, use of the space, and variations in bacterial composition to which newborns are exposed at birth.

Compared with previous studies depicting the microbial diversity in the OR, this study is based in an intensive sample of the four walls of one single room (N = 131), adding to intensive sample work by others in hospital settings [34]. This sampling method allowed us to make comparisons between samples and establish potential variables that seem to covariate with the sampling location and establish a relationship in which the variation can be explained as a function of the height of the sampling site. By characterizing those different groups, and connecting to height, we were able to relate the distribution of the different sampling points and the proportional occurrence of the most represented bacteria. Besides the similarity between the horizontal samples, variations mostly occur vertically with wall height. These significant differences in bacterial diversity and structure, with wall height, indicate that floor bacteria include fecal and soil associated anaerobes, while those that are more dominant in top areas are more aerophilic and skin related. Decreasing values in alpha diversity associated with walls' height and the discriminant taxa showing these differences seem to shed from the patients being intervened at the OR, including the gut and vaginal bacteria, while skin bacteria and aerobic bacteria were more dominant in the upper walls. This distribution suggests human sources, and perhaps reflects the density of bacteria-carrying particles (eg. feces vs skin flakes). The associations deserve further studies associating microbes in the wall surface to human and environmental pollutants, even if shoes are microbial sinks that colonize the built environment, as suggested in another study [35].

Air convection currents that circulate resident air from the floor up to the ceiling as a consequence of air conditioning [36], although we have not measured convection, it may play a significant role on distributing airborne and human skin bacteria in ways independent from transfer by direct contact [37]. Supporting these, our previous study used SourceTracker analysis showing that OR microbes could play a role in seeding infants born by C-section [5]. Babies born via C-section may solely be receiving this inoculum—causing significant alterations in the establishment of microbiota-, while vaginally born infants have exposure to vaginal bacteria such as *Lactobacillus* spp., such as *L*. *crispatus*, *L*. *gasseri*, *L*. *jensenii*, and *L*. *iners* [9]. As Parvizy [12] remarks, although the pharmaceutic and computer industries enforce stringent air quality standards on their manufacturing processes, there are currently no U.S. standards for acceptable air quality within the OR environment [38]. Indeed, there are regulations for temperature, relative humidity, pressure relationships, face velocity at the grilles and air-change rates, but still there is "no national standard for measuring the number of particles nor the number of colony-forming units (CFUs) in ORs" [2]. The results of this work help understand the possible source of the primordial bacterial exposure in C-section born babies, which lack the labor-acquired exposure, who are exposed to mostly human skin-soil bacteria in the environment of the OR. Birth implies a sudden exposure to environmental antigens, many of them derived from the operating room environment and human commensal bacteria. This leads to immediate innate and adaptive immune changes in response to the massive triggers to the baby's immune system. As the first line of defense against pathogens, the innate immune system composed by neutrophils, monocytes, macrophages and dendritic cells interact with the adaptive immune system. These cells mature at different times and in the newborn have a weak development. Certainly, the bacteria to which the babies' are exposed in the OR will in turn result in poor secretion of bioactive molecules and thus altered immune profiles, although any specific immune response-related the OR bacteria can only be mere speculations. To understand the potential connections between operating room bacterial exposure and increased immune risk in newborns, new studies are needed to clarify whether it is the lack of the normal maternal bacteria acquired during labor and birth [16] or the exposure to the OR soil/human skin-like bacteria first, what leads to increased disease risk. Microbiome restoration experiments will grant answers to these questions. In any case, we know that the OR is an artificial environment, and C-section by itself greatly disturbs the vertical -inter-generational- transmission of the primordial inoculum that has occurred in the matrilineal line of that baby [39] during the thousand generations in that baby ancestry. The widely accepted built space microbiota have differential effects on the occurrence of microorganisms on the surfaces of those spaces [40,41], and could be related to the occupant’s health. Therefore, little is known about how microbial dynamics vary across surfaces in a single physical space and their characteristics. Our intensive sampling of a single room at an OR shows a complex bacterial composition distributed across the walls of the ORs, that deserves characterization.

## Supporting information

S1 Table (XLSX)

S1 Data (PDF)
