# Supplementary material for: An in-depth survey of the microbial landscape of the walls of a neonatal operating room
Source: PLoS One. 2020 Apr 3;15(4):e0230957. doi: 10.1371/journal.pone.0230957 (PMC7122808; doi:10.1371/journal.pone.0230957)
Supplement: S1 Data — (PDF) [file pone.0230957.s002.pdf]

Supplementary Materials

Table S1. Pairwise comparison for permutation test of homogeneity of multivariate dispersions (PERMDISP) on beta diversity Bray-Curtis distances (Observed p-value below diagonal, permuted p-value above diagonal).

	**0cm**	**30cm**	**90cm**	**150cm**
**0cm**		**1.00E-03**	**0.001**	**1.00E-03**
**30cm**	**9.05E-08**		0.327	5.97E-01
**90cm**	**2.59E-11**	3.38E-01		7.32E-02
**150cm**	**9.50E-09**	5.83E-01	0.089	

Table S2. Number of reads and OTUs for each dust sample

Sample ID	Distance to Floor	Number of Reads	Number of OTUs
1.A1	0cm	13772	539
1.A2	30cm	12415	395
1.A3	90cm	9815	308
1.A4	150cm	7823	236
1.B1	0cm	11526	455
1.B2	30cm	9658	357
1.B3	90cm	10304	327
1.B4	150cm	7000	241
1.C1	0cm	20949	640
1.C2	30cm	14772	410
1.C3	90cm	13727	343
1.D1	0cm	5167	294
1.D2	30cm	12646	356
1.D3	90cm	4574	292
1.D4	150cm	2752	151
1.F1	0cm	21970	586
1.F2	30cm	2973	155
1.F3	90cm	5570	166
1.F4	150cm	2432	95
1.G1	0cm	31304	964
1.G2	30cm	4896	163
1.G3	90cm	2956	98
1.G4	150cm	2904	111
1.H1	0cm	20029	638
1.H2	30cm	6352	258
1.H3	90cm	1724	107
1.H4	150cm	5773	174
1.I1	0cm	18983	643
1.I2	30cm	17075	680
1.I3	90cm	7995	393
1.I4	150cm	6033	209
1.j1	0cm	22737	633
1.j2	30cm	4645	151
1.j3	90cm	10074	395
1.j4	150cm	3926	160
2.A1	0cm	29540	987

Sample ID	Distance to Floor	Number of Reads	Number of OTUs
2.A2	30cm	16789	339
2.A3	90cm	3799	223
2.A4	150cm	5117	167
2.B1	0cm	15582	757
2.B2	30cm	3180	250
2.B3	90cm	8972	297
2.B4	150cm	2347	402
2.C1	0cm	7909	539
2.C2	30cm	15246	290
2.C3	90cm	7040	97
2.C4	150cm	1987	699
2.D1	0cm	17356	606
2.D2	30cm	15629	191
2.D3	90cm	3729	86
2.D4	150cm	2489	1002
2.F1	0cm	18877	387
2.F2	30cm	13005	304
2.F3	90cm	9300	204
2.F4	150cm	6322	416
2.G1	0cm	13334	463
2.G2	30cm	23116	318
2.G3	90cm	8584	225
2.G4	150cm	6049	409
2.H1	0cm	10041	377
2.H2	30cm	13753	209
2.H3	90cm	5690	368
2.H4	150cm	12430	516
2.I1	0cm	17151	198
2.I2	30cm	4870	364
2.I3	90cm	10617	162
2.I4	150cm	4883	197
2.j1	0cm	2874	312
2.j2	30cm	13474	151
2.j3	90cm	3326	176
2.j4	150cm	6093	550
3.A1	0cm	24092	197
3.A2	30cm	8417	196

Sample ID	Distance to Floor	Number of Reads	Number of OTUs
3.A3	90cm	5251	186
3.A4	150cm	7581	350
3.B1	0cm	10638	204
3.B2	30cm	5270	222
3.B3	90cm	6898	229
3.B4	150cm	6180	370
3.C1	0cm	15262	294
3.C2	30cm	8494	235
3.C3	90cm	9137	152
3.C4	150cm	5115	299
3.D1	0cm	8140	517
3.D2	30cm	19045	115
3.D4	90cm	10122	235
3.F1	150cm	20119	544
3.F2	0cm	30653	494
3.F3	30cm	22134	485
3.F4	90cm	12344	324
3.G1	150cm	22006	797
3.G2	0cm	19914	487
3.G4	30cm	10703	275
3.H1	150cm	9005	437
3.H2	0cm	17951	460
3.H3	30cm	2839	121
3.H4	90cm	4711	159
3.I1	150cm	20757	432
3.I2	0cm	29517	671
3.I3	30cm	20395	469
3.I4	90cm	14970	273
3.j1	150cm	13492	348
3.j2	0cm	8298	259
3.j3	30cm	9703	206
3.j4	90cm	7033	146
4.A1	150cm	14982	362
4.A2	0cm	2481	131
4.A3	30cm	11188	235
4.A4	90cm	8732	185
4.B1	150cm	11593	408
4.B2	0cm	5743	177

Sample ID	Distance to Floor	Number of Reads	Number of OTUs
4.B3	30cm	5964	161
4.B4	90cm	7225	183
4.C1	150cm	13229	415
4.C2	0cm	23554	487
4.C3	30cm	7685	188
4.C4	90cm	9929	258
4.D1	150cm	17687	540
4.D2	0cm	9028	274
4.D3	30cm	8139	225
4.D4	90cm	6171	247
4.H4	150cm	7838	330
4.I1	150cm	11022	730
4.I2	0cm	24131	667
4.I3	30cm	6163	263
4.I4	90cm	8781	290
4.j1	150cm	32734	1006
4.j2	0cm	15112	411
4.j3	30cm	12426	524
4.j4	90cm	3344	172
3.D3	150cm	1492	85

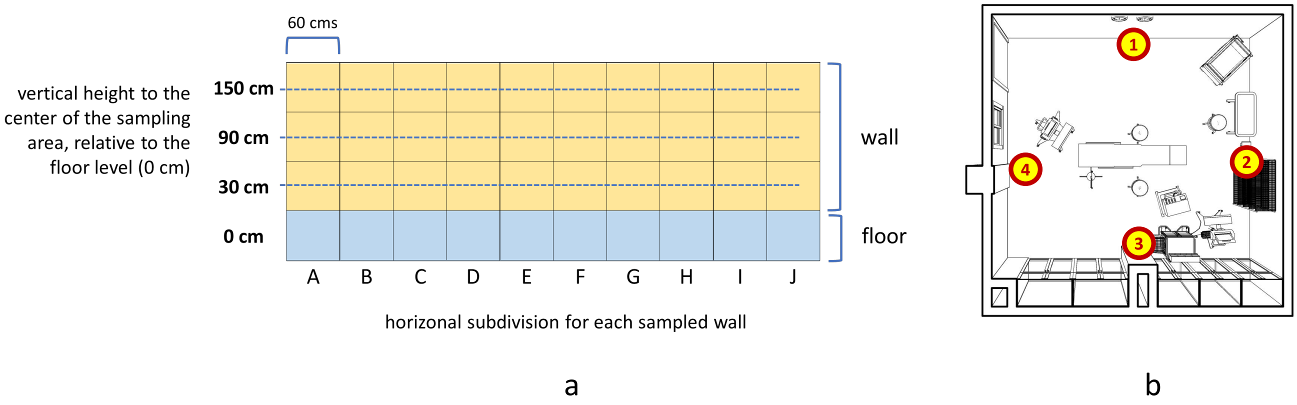

Figure S1: Panel A shows the distribution of sampling areas for each wall. Panel be shows a top view of of the operating room showing the sampled walls by wall number.

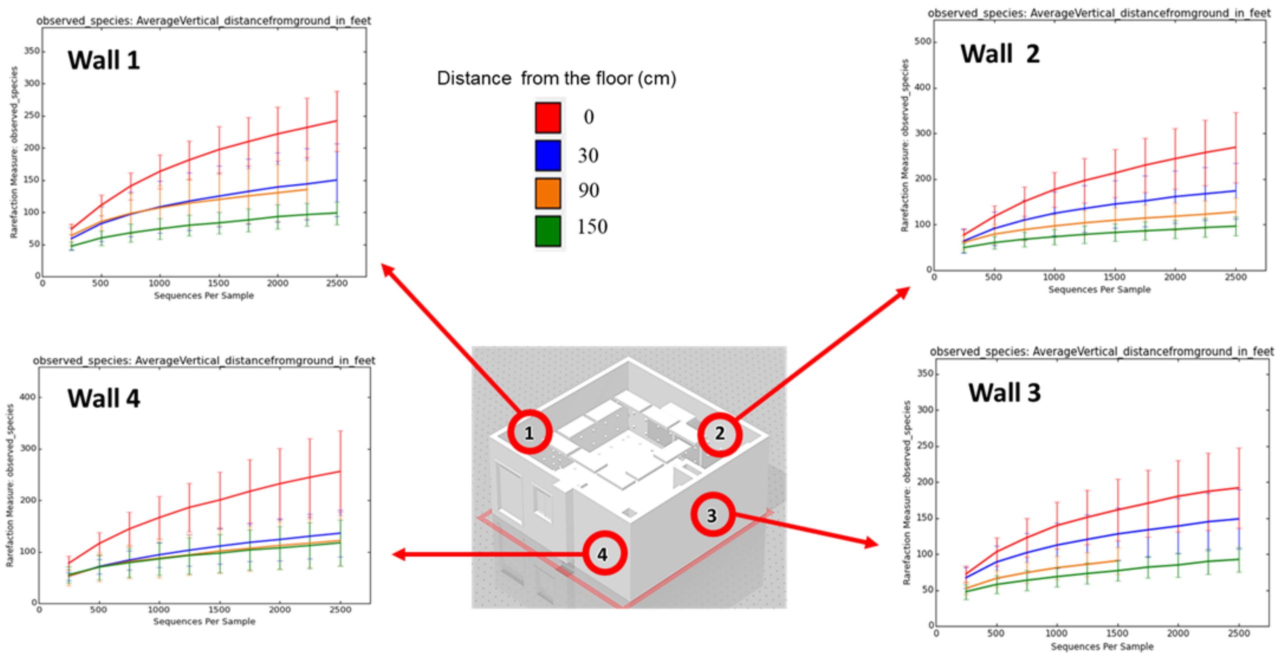

Figure S2. Alpha diversity at the different sampling heights, by sampled wall. It is possible to observe that species richness decrease with sampling height consistently for the four OR walls.

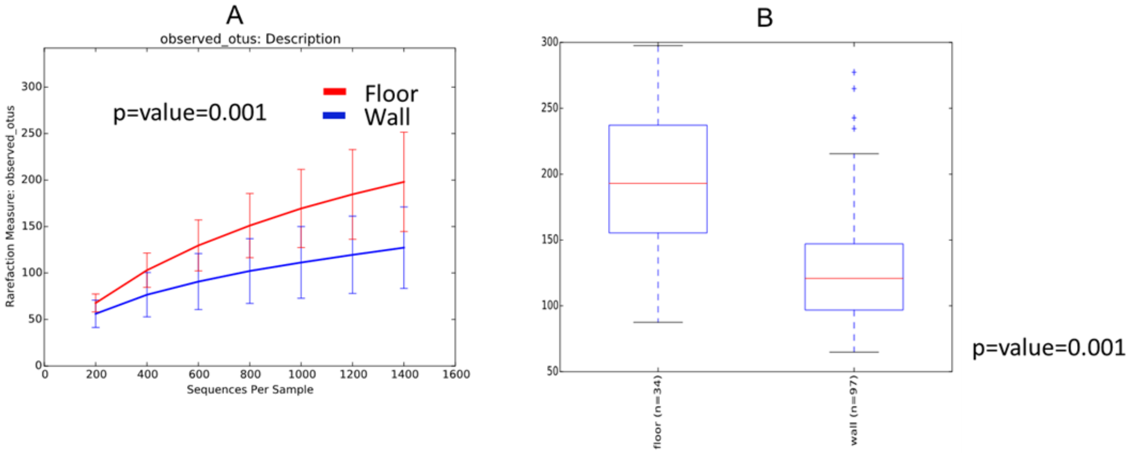

Figure S3. Alpha diversity of bacterial communities in the combined floor (0 cm level) and all walls (30,90,150cm) for the four OR walls. Panel A shows rarefaction curves of observed species, and Panel B presents boxplot sshowing species richness between floor-level and wall heights.
